# Supplementary material for: The Effect of Statin on Anemia in Patients with Chronic Kidney Disease and End-Stage Kidney Disease: A Systematic Review and Meta-Analysis
Source: J Pers Med. 2022 Jul 19;12(7):1175. doi: 10.3390/jpm12071175 (PMC9317421; doi:10.3390/jpm12071175)
Supplement: Supplementary file 1 [file jpm-12-01175-s001.zip › Key words_Mesh terms .pdf]

Supplementary Document S2: Key words.

Pubmed

1. (((dialysis) OR ESRD) OR chronic kidney disease) OR kidney failure
2. ((((((HMG-CoA reductase inhibitor) OR statin) OR atorvastatin) OR rosuvastatin) OR lovastatin) OR pravastatin) OR simvastatin
3. (((erythropoietin) OR recombinant erythropoietin) OR continuous erythropoiesis receptor activator) OR ESA
4. ((resistance) OR hyporesponsiveness) OR response
5. ((((erythropoietin resistance index) OR erythropoietin requirement) OR erythropoietin dose) OR hemoglobin) OR erythropoietin to hematocrit ratio

- Combination: #1 AND #2 AND (#3 OR #4 OR #5)

Medline

1. (((dialysis) OR ESRD) OR chronic kidney disease) OR kidney failure
2. ((((((HMG-CoA reductase inhibitor) OR statin) OR atorvastatin) OR rosuvastatin) OR lovastatin) OR pravastatin) OR simvastatin
3. (((erythropoietin) OR recombinant erythropoietin) OR continuous erythropoiesis receptor activator) OR ESA
4. ((resistance) OR hyporesponsiveness) OR response
5. ((((erythropoietin resistance index) OR erythropoietin requirement) OR erythropoietin dose) OR hemoglobin) OR erythropoietin to hematocrit ratio

- Combination: #1 AND #2 AND (#3 OR #4 OR #5)

Embase

1. 'hemodialysis'/exp OR hemodialysis OR 'peritoneal dialysis'/exp OR 'peritoneal dialysis' OR 'dialysis'/exp OR dialysis OR 'esrd'/exp OR esrd OR 'chronic kidney disease'/exp OR 'chronic kidney disease' OR 'kidney failure'/exp OR 'kidney failure'
2. 'hydroxymethylglutaryl coenzyme a reductase inhibitor'/exp OR 'atorvastatin'/exp OR 'rosuvastatin'/exp OR 'mevinolin'/exp OR 'pravastatin'/exp OR 'simvastatin'/exp
3. 'erythropoietin'/exp OR 'recombinant erythropoietin'/exp OR 'continuous erythropoiesis receptor activator'/exp OR 'erythropoietin stimulating agent'/exp OR esa OR 'recombinant erythropoietin' OR 'erythropoietin stimulating agent'
4. 'drug resistance'/exp OR resistance OR hyporesponsiveness OR response
5. 'erythropoietin resistance index' OR 'erythropoietin requirements' OR 'erythropoietin dose' OR ehr

- Combination: #1 AND #2 AND (#3 OR #4 OR #5)

Cochrane library

1. '[Renal Dialysis]'/exp OR '(dialysis):ti,ab,kw OR ("end stage renal disease"):ti,ab,kw OR (chronic kidney disease):ti,ab,kw OR (kidney failure):ti,ab,kw
2. '[Hydroxymethylglutaryl-CoA Reductase Inhibitors]'/exp OR 'atorvastatin'/exp OR 'rosuvastatin calcium'/exp OR 'lovastatin'/exp OR 'pravastatin'/exp OR 'simvastatin'/exp
3. 'erythropoietin'/exp OR 'epoetin alfa'/exp OR (continuous erythropoiesis receptor activator):ti,ab,kw OR (erythropoietin stimulating agent):ti,ab,kw OR ("recombinant erythropoietin"):ti,ab,kw
4. [Drug Resistance]/exp OR (hyporesponsiveness):ti,ab,kw OR (response):ti,ab,kw
5. (erythropoietin resistance index):ti,ab,kw OR (erythropoietin requirement):ti,ab,kw OR (erythropoietin dose):ti,ab,kw OR (erythropoietin to hematocrit ratio):ti,ab,kw OR (hemoglobin):ti,ab,kw

- Combination: #1 AND #2 AND (#3 OR #4 OR #5)
