# Supplementary material for: The Effect of Statin on Anemia in Patients with Chronic Kidney Disease and End-Stage Kidney Disease: A Systematic Review and Meta-Analysis
Source: J Pers Med. 2022 Jul 19;12(7):1175. doi: 10.3390/jpm12071175 (PMC9317421; doi:10.3390/jpm12071175)

Leave-One-Out Analysis

Figure S1:**Hemoglobin -- Baseline & Endpoint**

The following result are the analysis of baseline and endpoint value of hemoglobin in statin group and control group after leaving Nand et al. out. The upper section of the figure is the baseline value of hemoglobin, whereas the lower section of the figure is the endpoint value of hemoglobin.

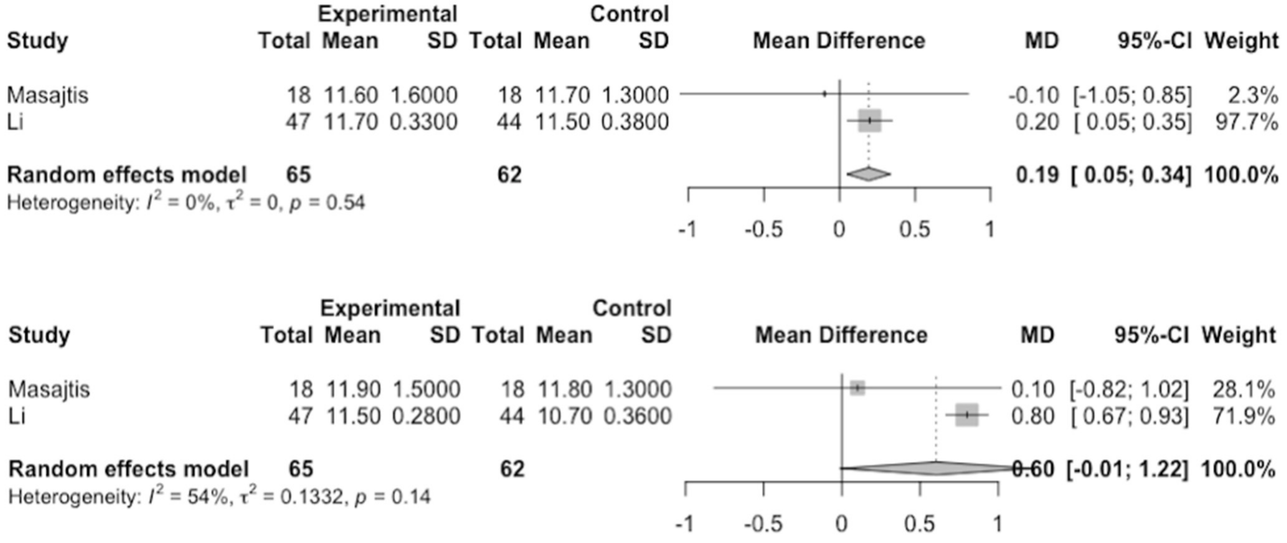

Figure S2: **Hemoglobin -- Single-arm**

The following result is the analysis of hemoglobin level in statin group solely after leaving Nand et al. and Sirken et al. out.

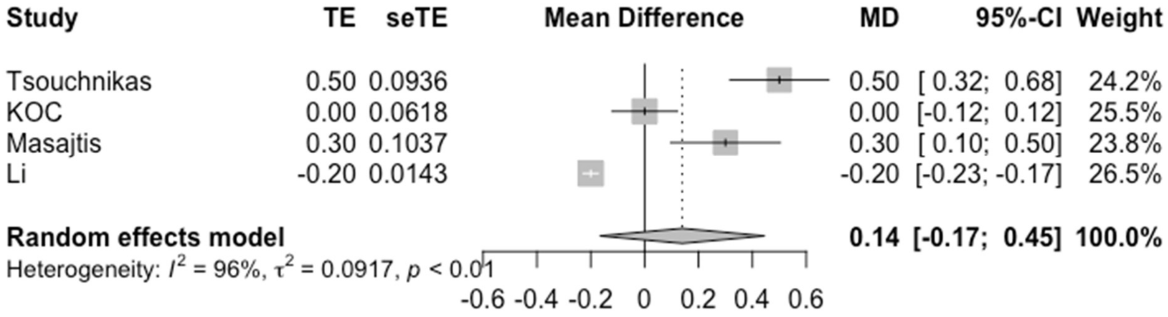

Figure S3: **Hemoglobin -- Change-from-baseline between statin group and control group**

The following result is the analysis of mean difference of change-from-baseline data of hemoglobin between statin group and control group after leaving Nand et al. out.

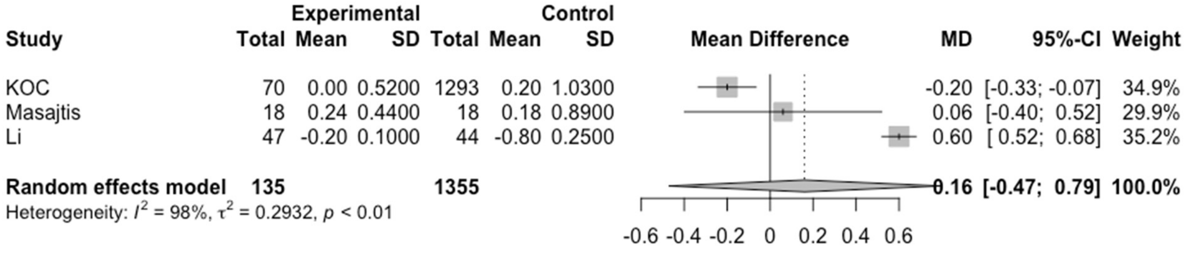

Supplement: Supplementary file 1 [file jpm-12-01175-s001.zip › Supplementary Figures.pdf]
